# Supplementary material for: The interactome of intact mitochondria by cross-linking mass spectrometry provides evidence for coexisting respiratory supercomplexes
Source: Mol Cell Proteomics. 2017 Dec 8;17(2):216–32. doi: 10.1074/mcp.RA117.000470 (PMC5795388; doi:10.1074/mcp.RA117.000470)
Supplement: Supplemental Data [file supp_RA117.000470_133922_0_supp_23978_fzffwf.zip › spectra_annotation/mito_spectra_annotation/252-1-5-1-3-1.pdf]

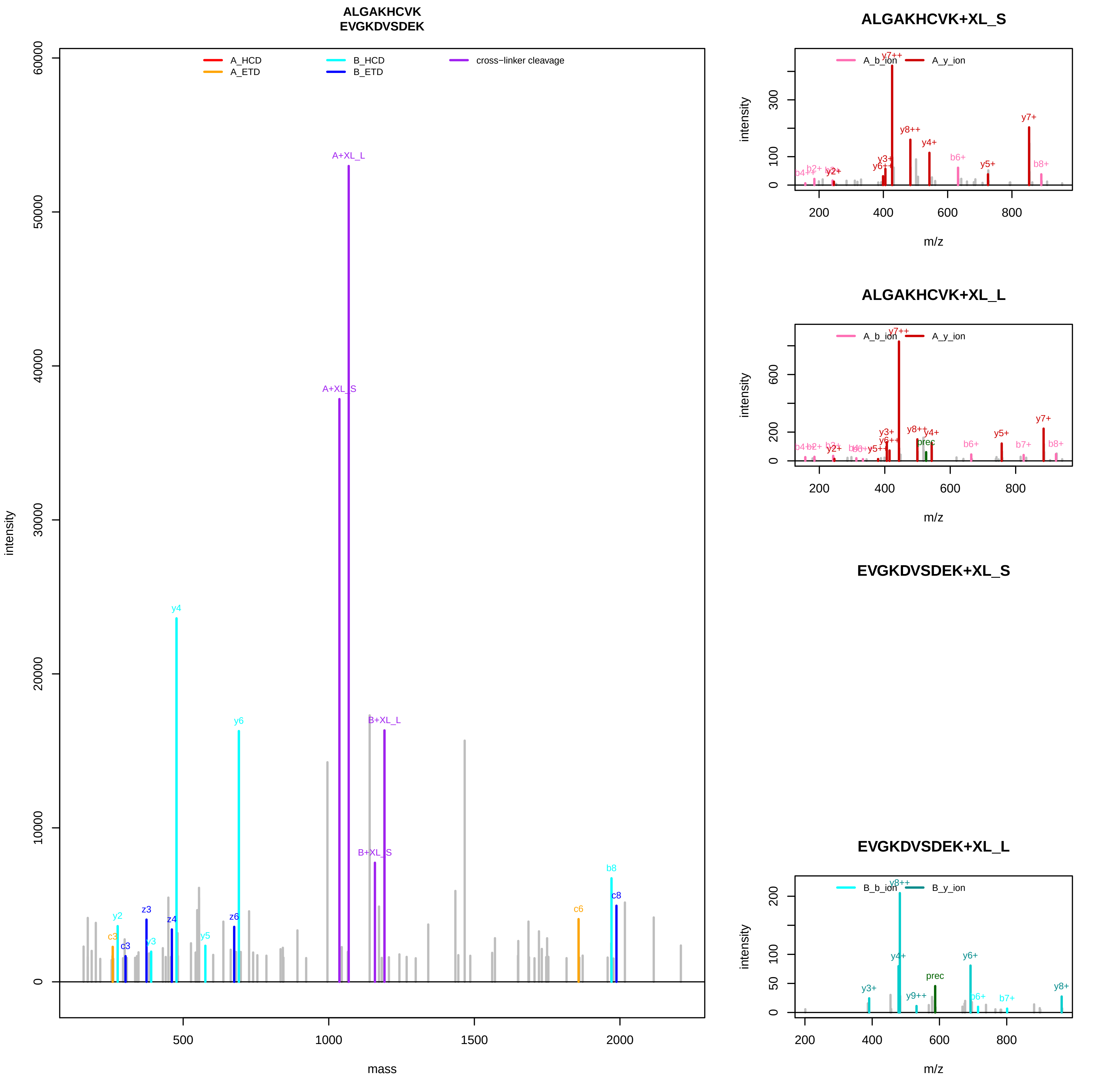
ALGAKHCVK
EVGKDVSDEK
A_HCD
A_ETD
B_HCD
B_ETD
cross−linker cleavage
A+XL_L
A+XL_S
B+XL_L
B+XL_S
y2
y3
y4
y5
y6
c3
c3
c6
c8
z3
z4
z6
b8
intensity
mass
0
10000
20000
30000
40000
50000
60000
500
1000
1500
2000
ALGAKHCVK+XL_S
ALGAKHCVK+XL_L
EVGKDVSDEK+XL_S
EVGKDVSDEK+XL_L
A_b_ion
A_y_ion
B_b_ion
B_y_ion
y7++
y8++
y7+
y4+
y5+
y3+
y6++
y2+
b2+
b6+
b7+
b8+
prec
y9++
b6+
b7+
y8+
y6+
m/z
200
400
600
800
